# Quality of counseling for self-administering injectable contraception: field evidence from mystery client interactions in Lagos, Nigeria

**DOI:** 10.1186/s12905-025-03946-2

**Published:** 2025-08-21

**Authors:** Sneha Challa, Calvin Chiu, Ayobambo Jegede, Ivan Idiodi, Mikail Aliyu, Chioma Okoli, Shakede Dimowo, Aminat Tijani, Awawu Grace Nmadu, Rodio Diallo, Jenny Liu, Elizabeth Omoluabi

**Affiliations:** 1 University of California, San Francisco, Institute for Health & Aging, School of Nursing, Box 0646, 490 Illinois St., 12th Floor, San Francisco, CA 94158 USA; 2 AkenaPlus Health, F Road #18, Citec Estate, Airport Road, Abuja, Nigeria; 3 University of California, Berkeley, School of Public Health, 2121 Berkeley Way, Berkeley, CA 94704 USA; 4 Bill and Melinda Gates Foundation, 500 5th Ave N, Seattle, WA 98109 USA

**Keywords:** Mystery clients, Family planning, Self-injection, Self-care, Nigeria, Quality of care

## Abstract

**Background:**

Self-injection (SI) of subcutaneous depot medroxyprogesterone acetate (DMPA-SC) is a self-care intervention (drugs, diagnostics, or devices that can be provided mostly outside the health system) implemented across Nigeria. Per national guidelines, first-time DMPA-SC users can obtain units for SI after two in-person training visits. Success of self-care interventions, the option for SI included, depends on local policies and individual providers to create an enabling environment. Thus, we aimed to 1) assess providers’ fidelity to Ministry of Health protocols; 2) assess the extent of bias in fidelity; and 3) to asses client-centeredness.

**Methods:**

Eight mystery client actors portrayed an older, married woman or a younger, unmarried woman without DMPA-SC experience. They sought contraception, including DMPA-SC for SI, at 30 public and 30 private facilities. A total of 120 interactions were planned (two per facility—one by each profile). Immediately following their interactions, actors completed a debrief survey about their experiences. Using responses from these debrief surveys, we described key actor-reported outcomes (providers’ fidelity to Ministry of Health protocols for SI dispensing, SI training, and supporting contraceptive decision-making). We also examined objective and subjective client-centeredness outcomes. We assessed bias in fidelity and client-centeredness through bivariate tests for differences by actor profile (younger/unmarried vs older/married) and facility type (health facility vs pharmacy/PPMV).

**Results:**

Fidelity to dispensing guidelines (i.e., refusing DMPA-SC units for SI) differed by facility type (χ^2^ = 12.4, *p*-value < 0.001). Descriptively, pharmacists/PPMVs more often broke with protocol and were willing to dispense DMPA-SC units. Similarly, fidelity to Ministry of Health training guidelines on DMPA-SC for SI differed by facility type (χ^2^ = 9.9, *p*-value = 0.007). Client-centeredness outcomes (e.g., being asked about and feeling treated differently based on age and marital status) were found to differ by actor profile. Descriptively, more of the younger, unmarried profile actors reported these outcomes compared to older, married profile actors.

**Conclusions:**

Willingness to dispense DMPA-SC for SI differed by facility type but not by client profile. However, younger, unmarried profile actors experienced more scrutiny from providers. These findings indicate a need for clarifying service provision protocols to ensure an enabling environment for women’s access to and use of self-injectable contraception.

**Supplementary Information:**

The online version contains supplementary material available at 10.1186/s12905-025-03946-2.

## Background

Subcutaneous depot medroxyprogesterone acetate (DMPA-SC; brand name Sayana Press) is an injectable contraceptive packaged in a prefilled syringe with a small needle, allowing for the option to self-inject by users. The option for self-injection (SI) is considered a self-care intervention–drugs, diagnostics, or devices that can be provided mostly outside the health system and are touted by the World Health Organization as critical to increasing health service coverage while alleviating burden on often strained health systems [1, 2]. Specifically, the SI option has potential to increase women’s autonomy by giving women who desire several months of protection from pregnancy the option for fewer health facility visits [3–5].

Demonstrating a commitment to implementing and scaling self-care interventions across the country, the Nigerian government introduced DMPA-SC in 2014, followed by a five-year DMPA-SC Accelerated Introduction and Scale-up Plan in 2017 [6, 7]. Since then, program implementers expanded contraceptive services to include DMPA-SC, and in some cases SI, into specific family planning projects in the private sector (through the IntegratE project), and in the public sector (through the RASuDiN project) [7–9]. In Nigeria, the private sector primarily comprises pharmacies and patent and proprietary medicine vendors (PPMVs) who sell pharmaceutical products but who do not have formal training in pharmacy while the public sector typically includes public health facilities (government primary health centers and clinics). The DMPA-SC implementation plan included a pilot period in a few states for offering the SI option to interested clients following a stepwise initiation process with prescribed provider oversight [7]. For the first DMPA-SC dose, providers should train clients and administer the injection. For the second dose (about three months later), providers are required to retrain the client and observe clients administering the injection themselves. After clients have successfully administered the dose with provider supervision, the client may be deemed eligible to take home units to self-inject subsequent doses. Determining eligibility requires, at minimum, the provider to evaluate whether the client competently performs four critical steps to self-inject: 1) mixing the solution by shaking vigorously for 30 s, 2) pushing the needle cap and port together to activate the device, 3) pinching the skin to form a “tent” and inserting the needle, and 4) squeezing the reservoir slowly, for 5 to 7 s, to inject the contraceptive [7]. For subsequent “refill” doses, providers should continue to confirm eligibility (i.e. check that clients are following appropriate procedures) before dispensing units to clients for SI [7].

To realize the full potential of SI, including its promise of increased self-determination, autonomy, and self-efficacy for clients, the WHO recognizes the importance of an enabling environment with robust capacity [1]. This requires that policies, programs, and providers support SI initiation for interested and capable clients. Thus, evaluation of programmatic success necessitates focusing on the distinct but interrelated implementation and service outcomes.^1^,^2^ Per Proctor et al. (2009 and 2011), implementation outcomes are “the effects of deliberate and purposive actions to implement new treatments, practices, and services” which represent key preconditions for attaining changes in service outcomes necessary for quality improvement” [10]. In the case of DMPA-SC for SI in Nigeria, the Innovations for Choice and Autonomy (ICAN) Consortium, a group of researchers focused on optimizing facilitators and reducing barriers to SI use, aimed to assess provision of SI as part of the full contraceptive method choice set. With support from implementing partner programs RASuDiN and IntegratE, the ICAN project conducted a mixed-methods implementation research study to assess the extent to which local policies and individual providers create an enabling environment for SI initiation by bolstering clients’ knowledge, skills, and self-efficacy.

Here, we present findings from mystery client (MC) interactions conducted with participating health facility and pharmacy/PPMV providers. These MC interactions simulated the experience of first-time clients seeking DMPA-SC units for the purposes of SI. The MC approach offers a standardized and objective way to measure de facto experiences, compared with provider- or consumer-based data which may involve reporting biases [11]. In this study, we have used MC interactions to assess providers’ actions and whether or not they were aligned with Ministry of Health protocols for quality contraceptive counseling provision. We focused on fidelity and client-centeredness, two key aspects of the Proctor et al. framework (Proctor, 2011) [10]. Fidelity refers to the extent to which an intervention is implemented as intended while client-centeredness describes the extent to which services are responsive to clients’ needs and preferences. In some cases, these two outcomes can be at odds, especially given that providers may have different motivations for offering their services. Maintaining appropriate client oversight and ensuring clients’ needs are met thus requires policies and programs to strike a balance. Our goals related to understanding this balance were threefold: 1) to assess providers’ fidelity to Ministry of Health protocols, (i.e., adherence to DMPA-SC for SI dispensing guidelines, adherence to DMPA-SC for SI training guidelines, and adherence to clinical protocols for supporting contraceptive decision-making); 2) to assess the extent of bias in adherence to DAMP-SC for SI protocols (i.e., differences in adherence based on client profile and facility type); and 3) to asses client-centeredness (i.e., by comparing objective and subjective perspectives on the provider–client interaction).

## Methods

### Sampling and data collection

In consultation with implementing partner programs, we selected three senatorial zones in Lagos, and within them, three local government areas (Alimosho, Ifako-Ijaiye, and Surulere). Implementing partner programs shared lists of participating facilities in each local government area where providers would have been trained to counsel on and offer DMPA-SC for SI. The sampling frame comprised 101 participating facilities (n_health facility_ = 62, n_pharmacy/PPMV_ = 39). After stratifying by local government area and health facility type, we randomly selected 60 facilities (30 per type with replacements) for MC interactions.

**Fig. 1:**
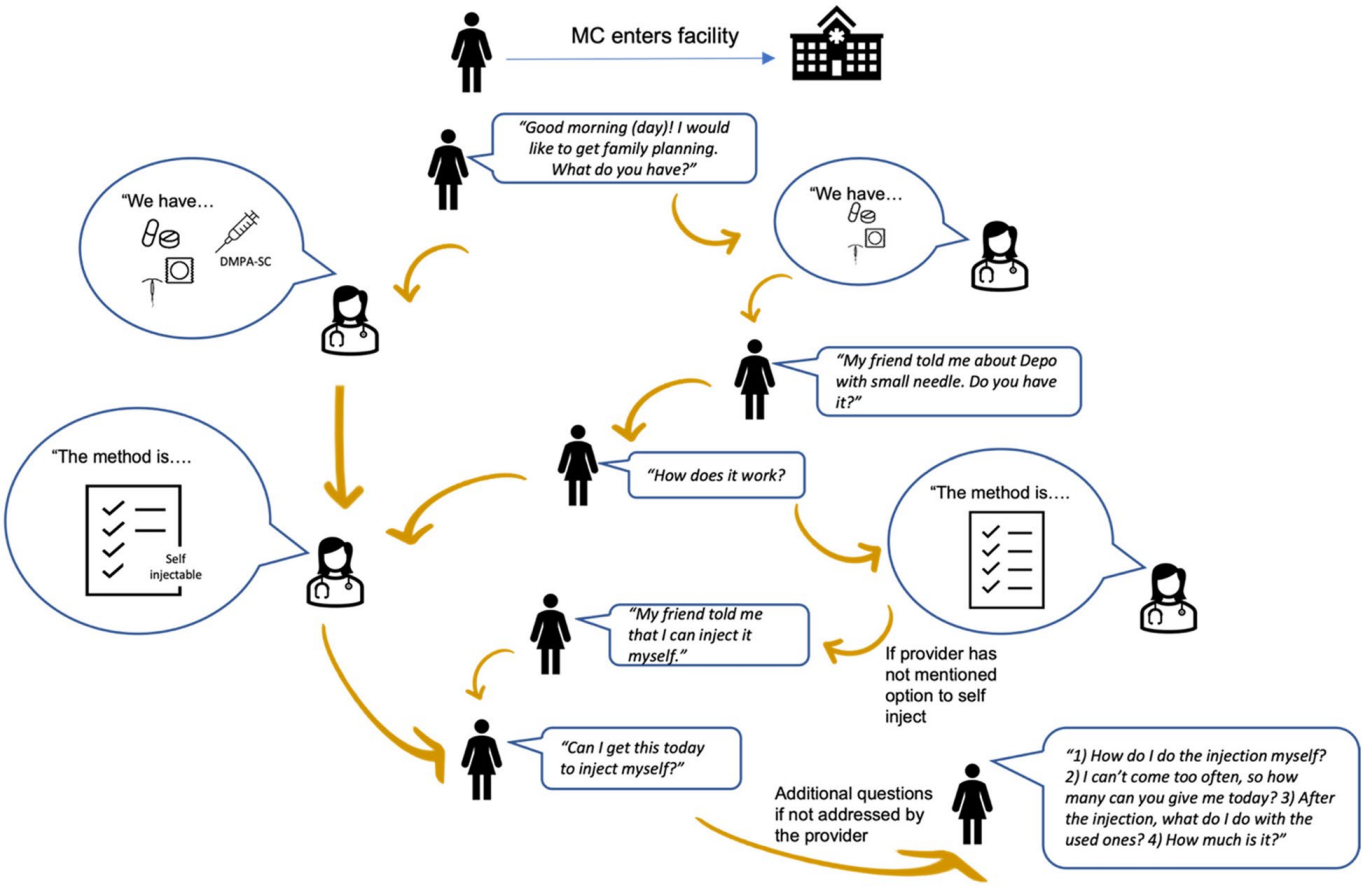
Flow of mystery client interaction for a first-time user of DMPA-SC seeking units for SI

Following previous simulated client studies, we hired and trained eight actors to portray two client profiles: (i) an 18-year-old, unmarried, sexually active woman, and (ii) a 31-year-old, married woman with three children [12]. A five-day training was implemented with all actors to ensure standardization of behavior during the interactions and reporting afterwards. In the training, actors learned about the MC methodology while memorizing their profile details and the standardized interaction script for entering a facility and expressing interest in contraception (Fig. 1). They were instructed to wear age-appropriate attire and present as typical women in the study areas. Actors were instructed not to divulge personal details about their profiles unless asked and to allow the conversation with providers to progress organically, prompting to ask specifically about DMPA-SC and the option for SI if not first mentioned by the provider. Training also included reviewing and practicing completing the post-interaction debrief questionnaire, which was programmed on ODK. Actors were trained to complete the questionnaire themselves on their mobile devices after the interaction in a location out of sight of the facility. After training, during a one-day pilot, actors tested the script and debrief questionnaire at nearby health facilities and pharmacies/PPMVs. After the pilot exercise, they regrouped with training facilitators to review pilot experiences and calibrate subjective responses on the post-interaction debrief questionnaire (see below). The standardized debrief questionnaire asked actors to report on: 1) the availability and cost of DMPA-SC for SI, 2) the quality and comprehensiveness of SI counseling, and 3) perception of treatment by the provider.

Interactions were conducted in November and December 2020. Each of the 60 selected facilities was visited by two actors, one per profile, for a total of 120 planned interactions. Actors were trained to allow the interaction to proceed naturally and only probe or prompt when certain information and options were not presented. They first asked generally what contraceptive methods were available. Then, if not mentioned naturally, they would ask for DMPA-SC or “Depo with small needle” as it is commonly known. If the provider did not mention the option for self-injection naturally, actors were trained to say they heard they could inject themselves. Interactions would continue based on what information providers offered with actors probing with questions such as, “How do I do the injection myself?” or “How many [units] can you give me today?” We did not require actors to complete their interaction by purchasing a unit and/or undergoing an injection procedure, so they were trained to say they would come back another time if providers were willing to dispense units. During the interaction actors noted what providers mentioned naturally compared to when they needed to probe, what (if any) other contraceptive methods were mentioned, and whether counseling elements were covered, as required by the Ministry of Health SI provision guidelines (“Measures” section) [7]. This method was successfully implemented in a previous MC exercise for DMPA-SC in Nigeria [12]. After receiving information from the provider and completing the interactions, actors said they would return at a later time to avoid undergoing an injection procedure (see Fig. 1). Providers were not informed of the visits beforehand to ensure their behavior remained natural. Actors saw a convenience sample of providers available at the time of their visit. It is possible that at smaller facilities, pharmacies, or PPMVs each of the two MC actors saw the same provider. However, at larger facilities this was not guaranteed so our analyses focused on the aggregate similarities/differences in actor experience and not on those within individual providers. To simulate a realistic random client experience while avoiding any suspicion that could bias the study, interactions were randomized by time of the day (morning vs. afternoon), which profile visited first (younger, unmarried or older, married), and which actor (among four per profile) conducted the visit.

### Measures

Our independent variables included actor profile (young, unmarried woman vs. married woman with children) and health facility type (health facilities vs. pharmacies/PPMVs). Our outcome measure across fidelity and client-centeredness are detailed in Table 1 and described below:

**Table 1:** Outcomes by quality category

Quality Dimension	Outcome	Definition
Fidelity		
* Primary Outcome*	Provider adherence to dispensation guidelines^*^	Did the provider agree to give you DMPA-SC for SI today? *Among those providers who had DMPA-SC units available
	Reasons for declining	1. Provider would need to train actor– any responses having to do with this being the clients first visit, the need for training on how to inject, etc 2. No stock of DMPA-SC– any responses having to do with limited availability of DMPA-SC 3. Provider doesn’t recommend SI– any responses indicating the provider does not feel SI is right for this client or that they don’t support SI in general 4. Only provider administered DMPA-SC is offered– any responses indicating that providers do not allow SI at this facility 5. Provider believes woman should use contraception after having children– any responses indicated providers discourage contraceptive use for nulliparous women or women who have not completed their families 6. Other
* Fidelity to Ministry of Health SI Counseling Guidelines* [7]	Counseling elements covered^*^	Sum of the number of required counseling elements covered Did the provider… 1. Explain how hormones affect the body? 2. Mention that the injection should be inserted at a downward angle? 3. Mention that when injecting, the port should touch the skin? 4. Mention that you should not massage the site after injecting? 5. Allow you to practice the injection technique on a model? 6. Inform you of when you should not self-inject? 7. Explain that fertility will return after discontinuation? 8. Give you counseling materials or resources? 9. Show you the instruction booklet? 10. Show you the calendar used to track reinjection dates? Show you additional injection units? *Among those providers who had DMPA-SC units available
* Fidelity to Clinical Protocols for Supporting Contraceptive Decision-making*	Provider asked why actor contraception	Did the provider ask about why you wanted contraception?
	Provider mentioned other contraceptive methods	Which other types of contraception (aside from DMPA-SC) did the provider mention? *coded as any vs. none
	Provider discussed side effects of other contraceptive methods	Did the provider discuss possible side effects for any of these other methods?
	Provider asked why acter wantedDMPA-SC	Did the provider ask about why you wanted DMPA-SC for SI?
	Provider discussed side effects of DMPA-SC	Did the provider describe side effects of DMPA-SC?
Client-centeredness		
* Objective*	Provider asked about age	Were you asked about your age?
	Provider asked about marital status	Were you asked about your marital status?
	Provider asked about number of children	Where you asked about how many children you have?
* Subjective*	Actor felt treated differently by age	Did you feel like you were treated differently because of your age?
	Actor felt treated differently by marital status	Did you feel like you were treated differently because of your marital status?
	Actor felt treated differently by number of children	Did you feel like you were treated differently because of how many children you have

1. Fidelity
  a Primary outcome: Fidelity to Ministry of Health DMPA-SC for SI dispensing guidelines: As a measure of fidelity, our primary outcome was whether providers followed guildelines for dispensing DMPA-SC units for SI. Since the Ministy of Health guidelines state that providers only send clients home with DMPA-SC units for SI after their second visit, demonstrating adherence meant providers should *not* have been willing to dispense DMPA-SC units for SI for clients presenting as first-time, naïve users of SI. This was a binary variable with 0 = yes adhered to guidelines (unwilling to dispense) and 1 = did not adhere to guidelines (willing to dispense). Measurement of this outcome was restricted to those with DMPA-SC units available to dispense.
  b Fidelity to Ministry of Health SI training guidelines was assessed by one outcome measure capturing how many of the 11 recommended SI counseling elements were covered (see Table 1 for the 11 elements) [7]. Actors’ dichotomous (yes/no) responses to individual items were summed then categorized as low, medium, or high at the 25th, 50th, and 75th percentile cutoffs respectively. Thus, 0 topics covered was categorized as “low” fidelity, one topic as “medium” fidelity and two or more topics as “high” fidelity. Like the primary outcome, analysis of this outcome was restricted to providers with DMPA-SC units available to dispense at the time of the interaction.
  c Fidelity to clinical protocols for supporting contraceptive decision-making was captured using key recommendations in the Nigerian family planning service provision protocols for questions to ask/information to give to clients when counseling on contraception [13]. Our analysis was conducted with five separate binary outcomes (1 = yes, 0 = no) corresponding to actors’ responses to whether or not the provider: 1) asked why they wanted contraception, 2) counseled on any methods other than DMPA-SC, 3) discussed side effects of any other methods, 4) asked why they wanted DMPA-SC for SI, and 5) mentioned side effects of DMPA-SC.

2) Client-centerednes
  a *Objective:* We used three objective measures of whether the provider asked questions about the actors’ personal characteristics (i.e., age, marital status, and number of children). Each outcome was treated as binary (1=yes, 0=no). These items have been used successfully as measures of client-centeredness in previous studies that showed providers treated young unmarried and older married clients differently [12].
  b *Subjective:* We used three subjective measures of actors’ perceptions of differential treatment based on age, marital status, and number of children. In MC actor training, “differential” was not given any negative or positive connotation. Instead, actors were trained to report on each outome reflecting whether the particular characteristic in question seemed to influence providers’ treatment of her. Each outcome was treated as binary (1=yes, 0=no). These measures have also been used successfully in previous studies in comparison to the above described objective outcomes [12].

### Analysis

We first described the types of facilities and providers visited. Next, we descriptively examined fidelity and client-centeredness (objective and subjective) outcomes. We assessed bias in fidelity to protocols and client-centeredness using bivariate (chi-squared tests) analyses to test for differences in the outcomes by actor profile and facility type. All analyses were conducted using Stata 16 (StataCorp, College Station, TX).

## Results

### Descriptive results

Of the 120 planned interactions, actors completed 117: 59 at health facilities facilities (29 younger, unmarried profile, 30 older, married profile) and 58 at pharmacies/PPMVs (29 younger, unmarried profile, 29 older, married profile). Actors reported a mean of 1.54 people waiting when they arrived with 59% reporting they waited less than 5 min. In the pharmacy/PPMV interactions, 67% reported waiting less than 5 min with a mean of 0.64 people waiting then they arrived compared to 51% waiting less than 5 min at health facilities and a mean of 2.45 people waiting. Most providers at health facilities were nurses/midwives, while those in the private facilities were predominantly pharmacists. Most providers were perceived by actors to be between the ages of 30 and 59 years and female (Table 2).

**Table 2:** Description of facilities and providers visited

	**Total number of facilities**	**Health Facilities n(%)**	**Pharmacies/PPMVs n(%)**	
Total	**117(100.0)**	**59(50.4)**	**58(49.6)**	*p*-value^*^
Provider Age				
Below 30	14(12.0)	3(5.1)	11(19.0)	0.06
30–39	33(28.2)	15(25.4)	18(31.0)	
40–49	40(34.2)	22(37.3)	18(31.0)	
50–59	29(24.8)	19(32.2)	10(17.2)	
60 and above	1(0.9)	0(0.0)	1(1.7)	
Provider Gender				
Female	96(82.1)	59(100.0)	37(63.8)	< 0.001
Male	21(18.0)	0(0.0)	21(36.2)	
Provider Type				
Other/Did not say	9(7.7)	4(6.8)	5(8.6)	< 0.001
Doctor	1(1.7)	2(3.4)	0(0.0)	
Nurse/midwife	58(49.3)	49(83.1)	9(15.5)	
JCHEW/CHEW^**^	5(4.3)	4(6.8)	1(1.7)	
Pharmacist	36(30.8)	0(0.0)	36(62.1)	
Chemist/drug shop	4(3.4)	0(0.0)	4(6.9)	
Other shop worker	3(2.6)	0(0.0)	3(5.2)	
Facility Type				
Pharmacy	53(44.9)	0(0.0)	53(91.4)	< 0.001
Chemist/drug shop	5(4.2)	0(0.0)	5(8.6)	
Government hospital/clinic/maternity	59(50.4)	59(100.0)	0(0.0)	
DMPA-SC Availability				
Available	86(76.8)	44(75.9)	42(77.8)	0.81
Not Available	26(23.1)	14(24.1)	12(22.2)	
Wait Times				
Less than 5 min	69(59.0)	30(50.9)	39(67.2)	0.05
5–15 min	24(20.5)	12(20.3)	12(20.7)	
15–30 min	16(13.7)	9(15.3)	7(12.1)	
31–60 min	3(2.6)	3(5.1)	0(0.0)	
1 h or more	5(4.3)	5(8.5)	0(0.0)	
	Mean(SD)	Mean(SD)	Mean(SD)	
People Waiting				
	1.5(3.6)	2.4(4.7)	0.6(1.7)	0.007
Cost per unit (when not free)^***^				
	717(263.8)	666.7(723.4)	720.5(223.4)	0.74

^*^p-value from chi-squared test for categorical variables and t-test for continuous variables

^**^JCHEW Junior Community Health Extension Worker, *CHEW* Community Health Extension Worker

^***^3 providers at health facilities quoted a non-zero charge to actors; 44 providers at pharmacies/PPMVs quoted a non-zero charge

Overall, in 112 interactions, providers (96%) knew of DMPA-SC and 86 (77%) had DMPA-SC units available, though 2 reported that units were expired. Of those interactions in which providers knew of DMPA-SC, 96% (*n* = 107) were with providers who knew it could be offered for SI. The median retail price per DMPA-SC unit quoted to actors was 700 Naira (~ $1.80 US per November 2020 exchange rate). Among the 76% (*n* = 44) of pharmacists/PPMVs who quoted a unit price, the median was 720 Naira while among the 5% of health facility providers (*n* = 3) who quoted a unit price, the median was 667 Naira, though it should be be provided for free at health facilities.

### Fidelity results

#### Primary outcome: provider fidelity to DMPA-SC dispensing guidelines

Of the 84 interactions in which providers had valid DMPA-SC units available, in 71% (*n* = 60) providers adhered to government dispensing guidelines and declined to give actors DMPA-SC units to take home at the end of their first self-infection counseling visit (Table 3). No difference by actor profile was found. However, there was a difference found by facility type (χ^2^ = 12.4, *p*-value < 0.001). In other words, there were differences between health facility providers and pharmacists/PPMVs in their willingness to dispense units to women without prior SI experience or training. In the interactions in which actors reported that providers had valid DMPA-SC units available but would not dispense any units to them, a need for training on SI was most often cited (52%) as the reason for maintaining fidelity to guidelines and refusing to dispense DMPA-SC units. No differences by actor profile or facility type were found in these reasons for refusals (results not shown).

**Table 3:** Bivariate associations of actor profile and facility type with fidelity to SI dispensing and counseling guidelines

	**Primary Outcome**		**Fidelity to Ministry of Health SI Training Guidelines**			
	Provider adherence to dispensation guidelines *N* = 84		Number of SI teaching elements covered (of 11)^*^N = 84			
	**N(%)**	χ^2^*p*-value	**Low**	**Medium**	**High**	χ^2^*p*-value
			**N(%)**	**N(%)**	**N(%)**	
Overall	60(71.4)		33(39.3)	21(25.0)	30(35.7)	
Actor Profile						
Married woman with children	28(71.8)	0.005 0.9	13(33.3)	10(25.6)	16(41.03)	1.2 0.5
Young, unmarried women	32(71.1)		20(44.4)	11(24.4)	14(31.1)	
Facility Type						
Health Facilities	38(88.4)	12.4 < 0.001	23(53.5)	11(25.6)	9(20.9)	9.9 0.007
Pharmacies/PPMVs	22(53.7)		10(24.4)	10(24.4)	21(51.2)	

Outcomes are conditional on availability of DMPA-SC units

^*^Outcome categories: low (0 counseling elements), medium (1 counseling element), high (2 or more counseling elements)

#### *Fidelity to ministry of health guidelines for SI counseling *

Among the 84 interactions in which providers had valid DMPA-SC units available, a mean of 1.6 (sd = 2.06) counseling elements of the total 11 recommended for DMPA-SC for SI outlined by Ministry of Health guidelines (Table 3) were covered [7]. Of the providers with valid DMPA-SC units available, 39% fell into the low fidelity category (0 elements covered), 25% fell into the medium category (1 element covered), and 36% fell into the high category (2 or more elements covered). In interactions with young, unmarried profile actors, the distribution was 44% low fidelity, 24% medium fidelity, and 31% high fidelity while in interactions with older, married profile actors, the distribution was 33% low fidelity, 25% medium fidelity, and 41% high fidelity. In health facility interactions, 53% were low fidelity, 26% were medium fidelity, and 21% were high fidelity, while in health facility interactions, 24% were low fidelity, 24% were medium, and 54% were high fidelity. While the level of fidelity to training guidelines was not found to differ by actor profile, there was evidence of difference by facility type (χ^2^ = 9.9, *p*-value = 0.007).

#### *Fidelity to clinical protocols for contraceptive decision-making *

In 22 interactions (19%), providers asked why actors wanted contraception, and in 94% of interactions, at least 1 method other than DMPA-SC was mentioned. A key clinical quality measure is that of side effects counseling, which actors reported both about DMPA-SC and other methods discussed. Of the 112 interactions in which providers knew of DMPA-SC, 30% included mention of any side effect of DMPA-SC while 27% included discussion of any side effect of the other contraceptive methods discussed. Additionally, providers asked actors why they wanted DMPA-SC in 13 interactions (11%). In bivariate associations, interactions in which providers asked why the actor wanted contraception or mentioned other contraceptive methods were not found to differ by actor profile or facility type. Supplementary material shows this in detail (See Table S1). However, interactions were found to differ by actor profile with respect to providers discussing side effects of DMPA-SC (χ^2^ = 4.1, *p*-value = 0.04).

### Client-centeredness results

#### Objective vs subjective

Among the objective client-centeredness outcomes, we found that number of children was the most frequently asked question (34% of interactions), followed by marital status (28% of interactions) and age (16% of interactions). Interactions in which providers asked about actors’ age (χ^2^ = 4.9, *p*-value = 0.03) and marital status (χ^2^ = 21.8, *p*-value < 0.001) were found to differ by actor profile, but no differences were found by facility type. Supplementary material shows these associations in more details (see Table S2). Similarly, among the subjective client-cetneredness outcomes, we found that actors most often felt treated differently by number of children (17% of interactions), followed by marital status (15% of interactions) and age (13% of interactions). Agaiinteractions in which actors felt treated differently by age (χ^2^ = 0.04, *p*-value = 0.003) and marital status (χ^2^ = 15.2, *p*-value < 0.001) were also found to differ by actor profile.

## Discussion

Using an MC methodology, we assessed fidelity and client-centeredness in provider–client interactions related to seeking DMPA-SC for SI. We examined fidelity across DMPA-SC dispensing guidelines, DMPA-SC for SI counseling guidelines, and clinical protocols for contraceptive decision-making, and tested for bias in fidelity between client profiles (older, married woman vs. younger, unmarried woman) and facility types (health facilities vs pharmacies/PPMVs). We also compared objective and subjective client experiences. In maintaining fidelity to the Nigerian Ministry of Health guidelines requiring at least two visits before dispensing DMPA-SC for at-home use, 71% of providers did not offer units of DMPA-SC for SI to actors portraying clients wishing to self-inject for the first time [7]. However, our findings indicate some deviations from dispensing guidelines as well as overall low fidelity to Ministry of Health Counseling guidelines. Levels of fidelity were found to differ by client profile and facility type across several outcomes while objective and subjective perceptions of the interactions were found to differ by client profile indicating that client-centered service provision may differ accordingly.

Our descriptive results uncovered that interactions with pharmacy/PPMV providers particularly diverged from guidelines for dispensing DMPA-SC for SI. In the pharmacy/PPMV interactions, there was less adherence to DMPA-SC dispensing guidelines (i.e., greater willingness to give actors DMPA-SC units to take home at the end of the interaction which would have been actors’ first visit for SI counseling). Bivariate analyses support this with evidence of significant differences in adherence by facility type. Pharmacists/PPMVs may be more likely to offer take-home units given the potential for earning revenue with each sale as compared to health facility services which are given ostensibly for free. Compared to a small proportion of health facility providers, most interactions with pharmacists/PPMVs included a median cost higher than the recommended retail price of 500 Naira (~ $1.30 US per November 2020 exchange rate) per unit [14]. Given these costs for services, barriers to contraceptive access may persist, particularly for those who cannot afford to make repeat visits to a provider for SI training or to pay the price pharmacists/PPMVs set for DMPA-SC units. At the same time, poviders may feel thwarted by these rigid guidelines that put them at risk of losing revenue and/or clients when they are unable to give clients what they want (e.g., units to take away). Importantly, the stepwise initiation process requiring multiple visits for counseling and training is a unique feature of the Nigerian DMPA-SC for SI policy. This is in comparison to other contexts, such as in Uganda and Malawi, where SI is supported from the first visit [3, 15]. Importantly, implementation frameworks suggest that interventions may take stronger root when they can be adapted to respond to specific contexts, conditions, and clients [10, 16]. Thus, if policies forego precise fidelity in favor of allowing providers to operate with some leeway, they may rate them as more acceptable, and be more motivated to offer services in ways that meet their clients needs.

Across facility types and actor profiles, there was low fidelity to the Ministry of Health SI counseling guidelines with interactions including coverage of few of the recommended SI counseling elements. Our study protocol did not require actors to complete their interaction by purchasing a unit and/or undergoing an injection procedure [7]. However, this should not have influenced providers’ initial counseling on the method and for SI. This is because the Nigeria family planning service protocols require providers to counsel on the method of interest and mention other methods briefly before the client makes a choice (i.e. before deciding to undergo the initial injection) [13]. Thus, we would expect all interactions to have included comprehensive counseling and training on SI of DMPA-SC because all facilities visited participated in either RASuDiN or IntegratE programs, through which providers would have been trained on contraceptive counseling, including for SI of DMPA-SC. In particular, pharmacists/PPMVs were found to offer slightly more comprehensive counseling, demonstrating higher fidelity to protocols than health facility providers. Bivariate results support this, indicating significant differences in fidelity to counseling guidelines by facility type. Research shows that many providers, particularly in health facilities, face time constraints due to high client volume [17, 18]. In fact, evaluation of SI delivery in Uganda similarly found that a lack of time to train clients compromised counseling quality [17]. This is echoed by our findings showing more people waiting and higher wait times in the health facilities vs pharmacies/PPMVs. Providers seen in pharmacy/PPMV interactions likely faced fewer time pressures to attend to many clients during times the actor arrived. This may have served to facilitate more comprehensive counseling. Provider training programs in Nigeria have already condensed the self-administration steps for DMPA-SC to make it easier to train clients [7]. However, our findings suggest that providers may benefit from additionally simplifying counseling guidelines (e.g., with a minimally sufficient checklist) and consolidating the SI initiation process to better facilitate both offering and use of SI.

Our examination of fidelity to clinical protocols for supporting contraceptive decision-making showed that side effects of DMPA-SC were discussed differently by actor profile. Descriptively, more interactions with younger, unmarried profile actors included these discussions compared to those with older, married profile actors. Counseling on side effects is specifically outlined in the family planning service protocols and is generally a part of comprehensive family planning counseling [13]. Discussion about side effects is a critical part of contraceptive counseling because clients’ concerns over side effects are a major reason for method discontinuation and non-use, including specifically DMPA-SC [19–21]. It is possible that providers assume younger clients have less contraceptive use experience and need more information on side effects. However, it is also possible that providers concerned about contraception-related infertility or “inappropriate” adolescent sexual activity may restrict access to contraception for younger and nulliparous clients. This is evidenced by previous research across sub-Saharan Africa demonstrating that providers impose restrictions on specific methods that are not justified not by any clinical protocol or contraindication. Instead providers’ operated based on their personal beliefs about the appropriatness of certain methods for certain clients [12, 22–24]. Thus the context, content, and intention behind discussions about side effects merit further exploration, particularly in interactions with younger, unmarried, or nulliparous clients. Future research should explore how providers describe their provision of SI services to different client profiles and also seek to understand clients’ perception and navigation of any provider imposed restrictions.

While fidelity is an important aspect of implementation, maintaining fidelity does not guarantee client-centerdness. Thus, we also compared objective and subjective client-centeredness outcomes. Descriptive and bivariate results showed that there were in fact significant differences across actor profiles across both objective and subjective outcomes (i.e., being asked about and feeling treated differently by age and marital status, respectively. Despite the assurance of clients’ right of, “access to obtain services regardless of age, sex, creed, colour, marital status, or location”, previous research in Nigeria has provided strong evidence of provider bias, including minimum age, marital status, and minimum parity restrictions [12, 23]. On the one hand, asking clients about themselves (e.g., their age and marital status) could be an attempt to get to know the client. However, it could also give the provider basis to impose their self-determined eligibility criteria for certain methods and administration routes (e.g., long-acting methods and self-injecting being more appropriate for older/married clients). Similarly, while being treated differently could be positive (e.g., more attentive counseling due to a clients’ inexperience), it could also have negative implications (e.g., dissuading clients from using particular methods because they don’t have children). While we did not ascribe negative or positive connotation to these outcomes, the fact that both objective and subjective outcomes differed by actor profile, provides reason to believe some level of provider bias by actor profile was at play. This dynamic may be especially true given the relative novelty of the SI option and providers’ reticence to believe certain clients (e.g., young people) would be capable of proper administration. While contraceptive self-care initiatives, including introduction and scale-up of DMPA-SC for SI, aim to increase women’s autonomy, touchpoints with the health system are still necessary. Thus, personal questions unrelated to clinical protocols and attitude shifts from providers may negatively affect the client experience. In the context of our study, young, unmarried profile actors, portraying women who are often deemed “ineligible” for certain contraceptive methods in this context, were not denied SI services outright, but faced extra scrutiny compared to older, married profile actors. Such negative experiences may limit the extent to which SI of DMPA-SC supports women who face the most barriers to achieve their goals.

Our findings should be interpreted considering several limitations. First, while the facility sampling frame was provided by implementing partner programs from their list of participating facilities, we could not guard against other untrained staff being the frontline dispenser (especially in the face of high turnover). Next, our sample size was limited by a number of providers not having DMPA-SC stock available to dispense, limiting the strength of the conclusions we can draw. Additionally, a known limitation of MC interactions is that of actors’ recall bias. However, we sought to minimize risk of recall bias by instructing actors to complete the debrief survey immediately after the interaction. Given our focus on self-injectable DMPA-SC, we solely measured providers’ stated willingness to give DMPA-SC units rather than actual product dispensing to protect actors from undergoing the injection procedure. Finally, our outcomes were all considered neutral, so results should be interpreted carefully. In practice, clients’ characteristics could change a providers’ approach to counseling (positively or negatively), and actors may have been primed to think more critically about their treatment due to their training. Thus, our goal was not to make a judgement about whether each outcome was good or bad but to objectively describe what occurred in the interactions and compare it to what should have happened. This gave an objective assessment of fidelity to service provision protocols with any bias in fidelity captured by testing for differences by actor profile and facility type. Because fidelity does not always equate with client-centeredness, we added the subjective client-centeredness outcomes to capture actors’ perception of their treatment. These were also intentionally kept neutral to avoid actors’ overinterpretation of providers’ intentions.

Our robust actor training, study design, and comprehensive debrief survey allow us to make important assessments of fidelity to service protocols and client-centered service provision to inform DMPA-SC for SI scale-up across Nigeria. Future research should examine provider and client perspectives side-by-side to understand feelings about how the regulatory environment and SI training programs can better support self-care initiatives, and the provision of and access to high-quality, unbiased counseling. The SI option is uniquely positioned to enable women to use contraception with supportive provider intervention while alleviating the burden on strained healthcare systems. While provider–client touchpoints remain necessary, during these interactions, providers may contribute to barriers that have long-influenced young, unmarried women’s access to contraception. Thus, we assert that requiring strict adherence to rigid service protocols, while critical to establish safety and feasibility during initial implementation periods, may negatively affect acceptability and client-centeredness in the longer term. It is possible that recommending a minimally sufficient set of DMPA-SC for SI counseling elements and relaxing the stepwise SI initiation process to allow providers to send clients home earlier would be beneficial. At the same time, more explicit and direct guidance for contraceptive service provision to marginalized groups (e.g., young people) and increasing supervision (e.g., through in-person visits or virtually) may improve provider performance and client satisfaction. These strategies go above and beyond simple training or values clarification exercises to acknowledge the broader ecosystem that influences the provider–client interaction. They may have the potential to shift focus from rote memorization of and fidelity to guidelines to equip providers to offer services in more client-centered manner.

## Conclusion

This study highlights important variations in the extent to which providers maintain fidelity and client-centeredness in counseling for SI of DMPA-SC by facility type and client profile in Lagos, Nigeria. These findings are particularly critical as provision of the novel method DMPA-SC is scaled across the country and more providers are trained to offer the option for SI. In particular, future research should focus on the experiences of younger, unmarried clients to understand how they can be better supported to achieve their contraceptive preferences. Additionally, the regulatory environment may benefit from increased efforts to structure service provision protocols to ensure an enabling environment for self-care to better meet the needs of both providers and clients.

## Supplementary Information

Additional file 1: Table S1. Bivariate associations of actor profile and facility type with fidelity to clinical protocols for contraceptive decision-making. Description of data: Table showing bivariate associations between between actor profile/facility type and fidelity to clinical protocols outcomes.

Additional file 2: Table S2. Bivariate associations of actor profile and facility type with subjective and objective client-centeredness outcomes. Description of data: Table showing bivariate associations between actor profile/facility type and client-centeredness outcomes.

## Data Availability

The dataset used and/or analyzed during the current study are available from the corresponding author on reasonable request.

## Abbreviations

DMPA-SC: Subcutaneous depot medroxyprogesterone acetate
SI: Self-injection
ICAN: Innovations for Choice and Autonomy
MC: Mystery client

## Footnotes

1 Implementation Outcomes: acceptability, adoption, appropriateness, costs, feasibility, fidelity, penetration, sustainability.

2 Service Outcomes: efficiency, safetly, effectiveness, equity, patient-centeredness, timeliness.
