# Supplementary material for: Quality of counseling for self-administering injectable contraception: field evidence from mystery client interactions in Lagos, Nigeria
Source: BMC Womens Health. 2025 Aug 21;25(Suppl 1):399. doi: 10.1186/s12905-025-03946-2 (PMC12369023; doi:10.1186/s12905-025-03946-2)
Supplement: Supplementary file 1 — Additional file 1: Table S1. Bivariate associations of actor profile and facility type with fidelity to clinical protocols for contraceptive decision-making. Description of data: Table showing bivariate associations between between actor profile/facility type and fidelity to clinical protocols outcomes. [file 12905_2025_3946_MOESM1_ESM.docx]

**Table S1. Bivariate associations of actor profile and facility type with fidelity to clinical protocols for contraceptive decision-making**

|  | **Provider asked why actor wanted contraception**  **N=117** | | **Provider mentioned other contraceptive methods***  **N=117** | | **Provider discussed side effects of other methods**  **N=117** | | **Provider asked why actor wanted DMPA-SC for SI**  **N=117** | | **Provider described side effects of DMPA-SC**  **N=112** | |
| --- | --- | --- | --- | --- | --- | --- | --- | --- | --- | --- |
|  | **N(%)** | **χ^2^**  **p-value** | **N(%)** | **χ^2^**  **p-value** | **N(%)** | **χ^2^**  **p-value** | **N(%)** | **χ^2^**  **p-value** | **N(%)** | **χ^2^**  **p-value** |
| **Overall** | 22(18.8) |  | 110(94.0) |  | 31(26.5) |  | 13(11.1) |  | 33(29.5) |  |
| **Actor Profile** |  |  |  |  |  |  |  |  |  |  |
| Married woman with children | 8(13.8) | 1.9  0.2 | 53(91.4) | 1.5  0.2 | 12(20.7) | 2.0  0.2 | 7(12.1) | 0.1  0.7 | 11(20.4) | 4.1  0.04 |
| Young, unmarried women | 14(23.7) |  | 57(96.6) |  | 19(32.2) |  | 6(10.2) |  | 22(37.9) |  |
| **Facility Type** |  |  |  |  |  |  |  |  |  |  |
| Public | 11(18.6) | 0.002  1.0 | 55(93.2) | 0.1  0.7 | 18(30.5) | 1.0  0.3 | 6(10.2) | 0.1  0.7 | 19(32.8) | 0.6  0.4 |
| Private | 11(20.0) |  | 55(94.8) |  | 13(22.4) |  | 7(12.1) |  | 14(25.9) |  |

*Outcome categories: any vs none
