# Supplementary material for: Quality of counseling for self-administering injectable contraception: field evidence from mystery client interactions in Lagos, Nigeria
Source: BMC Womens Health. 2025 Aug 21;25(Suppl 1):399. doi: 10.1186/s12905-025-03946-2 (PMC12369023; doi:10.1186/s12905-025-03946-2)
Supplement: Supplementary file 2 — Additional file 2: Table S2. Bivariate associations of actor profile and facility type with subjective and objective client-centeredness outcomes. Description of data: Table showing bivariate associations between actor profile/facility type and client-centeredness outcomes. [file 12905_2025_3946_MOESM2_ESM.docx]

**Table S2. Bivariate associations of actor profile and facility type with subjective and objective client-centeredness outcomes**

| **Panel A: Objective Outcomes** | | | | | | |
| --- | --- | --- | --- | --- | --- | --- |
|  | **Provider asked about your age?**  **N=117** | | **Provider asked about marital status?**  **N=117** | | **Provider asked about number of children?**  **N=117** | |
|  | **N(%)** | **χ^2^**  **p-value** | **N(%)** | **χ^2^**  **p-value** | **N(%)** | **χ^2^**  **p-value** |
| **Overall** | 19(16.1) |  | 33(28.0) |  | 40(33.9) | N=40 |
| **Actor Profile** |  |  |  |  |  |  |
| Married woman with children | 5(8.6) | 4.9  0.03 | 5(8.6) | 21.8  <0.001 | 16(27.6) | 2.2  0.1 |
| Young, unmarried women | 14(23.7) |  | 28(47.5) |  | 24(40.7) |  |
| **Facility Type** |  |  |  |  |  |  |
| Public Type | 11(18.6) | 0.5  0.5 | 17(28.8) | 0.02  0.9 | 22(37.3) | 0.5  0.5 |
| Private Type | 8(13.8) |  | 16(27.6) |  | 18(31.0) |  |
| **Panel B: Subjective Outcomes** | | | | | | |
|  | **Actor felt treated differently because of your age**  **N=117** | | **Actor felt treated differently because of your marital status**  **N=117** | | **Actor felt treated differently because of your number of children**  **N=117** | |
|  | **N(%)** | **χ^2^**  **p-value** | **N(%)** | **χ^2^**  **p-value** | **N(%)** | **χ^2^**  **p-value** |
| **Overall** | 15(12.8) |  | 17(14.5) |  | 20(17.1) |  |
| **Actor Profile** |  |  |  |  |  |  |
| Married woman with children | 2(3.5) | 0.04  0.003 | 1(1.7) | 15.2  <0.001 | 11(19.0) | 0.3  0.6 |
| Young, unmarried women | 13(22.0) |  | 16(27.1) |  | 9(15.3) |  |
| **Facility Type** |  |  |  |  |  |  |
| Public Type | 9(15.3) | 0.6  0.4 | 8(13.6) | 0.09  0.8 | 8(13.6) | 1.04  0.3 |
| Private Type | 6(10.3) |  | 9(15.5) |  | 12(20.7) |  |
